# Results from Ireland North and South’s 2022 report card on physical activity for children and adolescents

**DOI:** 10.1016/j.jesf.2023.12.003

**Published:** 2023-12-09

**Authors:** Angela Carlin, Sinead Connolly, Tamsyn Redpath, Sarajane Belton, Tara Coppinger, Conor Cunningham, Alan Donnelly, Kieran Dowd, Deirdre Harrington, Elaine Murtagh, Kwok Ng, Wesley O'Brien, Lauren Rodriguez, Catherine Woods, Helen McAvoy, Marie Murphy

**Affiliations:** a Centre for Exercise Medicine, Physical Activity and Health, School of Sport, Ulster University, Belfast, UK; b School of Health and Human Performance, Dublin City University, Dublin 9, Ireland; c Department of Sport, Leisure & Childhood Studies, Munster Technological University, Cork, Ireland; d School of Medicine Faculty of Life and Health Sciences, Ulster University, Belfast, UK; e Physical Activity for Health Research Centre, Health Research Institute, University of Limerick, Limerick, V94 T9PX, Ireland; f Department of Sport and Health Sciences, Technological University of the Shannon, Athlone, Westmeath, Ireland; g Psychological Sciences and Health, University of Strathclyde, Glasgow, Scotland, UK; h Faculty of Education, University of Turku, Rauma, Finland; i School of Educational Sciences and Psychology, University of Eastern Finland, Joensuu, 80101, Finland; j School of Education, University College Cork, Cork, Ireland; k Institute of Public Health, Dublin, Ireland; l Physical Activity for Health Research Centre, Institute of Sport Physical Education and Health Sciences, Moray House School of Education & Sport, UK

**Keywords:** Sport, Sedentary behaviour, Surveillance, Physical education, Active transport

## Abstract

**Background:**

The Ireland North and South Report Card on Physical Activity (PA) for Children and Adolescents aims to monitor progress in PA participation across a range of internationally established indicators.

**Methods:**

Data were collated for 11 indicators and graded following the harmonised Active Healthy Kids Global Alliance report card process. Six representative studies (sample size range n = 898 to n = 15,557) were primarily used in the grading, with many indicators supplemented with additional studies and reports. Data collected since the implementation of COVID-19 public health measures in March 2020 were excluded.

**Results:**

Grades were awarded as follows: ‘Overall physical activity’, C-; ‘Organised Sport and Physical Activity’, C; ‘Active Play’, INC; ‘Sedentary Behaviours’, C-; ‘Physical Fitness’, INC; ‘Family and Peers’, D+; ‘School’, C-; ‘Physical Education’, D; ‘Community and Environment’, B+ and ‘Government’, B. Separate grades were awarded for disability as follows; ‘Overall physical activity’, F; ‘Organised Sport and Physical Activity’, D; ‘Sedentary Behaviours’, C-; ‘Family and Peers’, C; ‘School’, C- and ‘Government’, B. ‘Active Play’, ‘Physical Fitness’, ‘Physical Education’ and ‘Community and Environment’ were all graded INC for disability. Since the last report card in 2016, four grades remained the same, three increased (‘Overall physical activity’, ‘School’ and ‘Physical Education’) and two (‘Family and Peers,’ and ‘Government’) were awarded grades for the first time.

**Conclusion:**

Grades specific to children and adolescents with disability were generally lower for each indicator. While small improvements have been shown across a few indicators, PA levels remain low across many indicators for children and adolescents.

## 1 Introduction

Globally, approximately 81 % of children and adolescents are not meeting physical activity (PA) guidelines.^1^ Concerns about levels of inactivity in children and adolescents have been reported in Ireland North and South for many years, with 87 % of youth not achieving the recommended levels of PA.^2^^,^^3^ Despite efforts to increase PA in this population, rates of physical inactivity remain high across the island of Ireland.^4^^,^^5^

Ireland and Northern Ireland are two separate jurisdictions on the same island. The 2016 Get Ireland Active! ‘National Physical Activity Plan’ for Ireland^6^ aims to increase the proportion of children undertaking at least 60 min of moderate-to-vigorous PA (MVPA), every day, by 1 % per year. In 2022, the ‘Active Living - Sport and Physical Activity Strategy for Northern Ireland’ was launched,^7^ with the goal of providing children and adolescents with ‘the best start in life through sport and PA opportunities’. Due to the political landscape and small size of the island, government agencies, stakeholders and researchers work cooperatively to progress PA surveillance activity and policy agendas. Notably, in 2014 and 2016, the Ireland North and South report cards were presented as a unified report on children's PA for the whole island.^8^^,^^9^ In 2018, data on children's sport participation and PA was collated on an all-island basis for the first time.^3^ In 2022, an All-Island Physical Literacy Consensus Statement was launched by Sport Ireland and Sport Northern Ireland and aims to encourage lifelong participation in sport and PA.^10^

There is much evidence to support the notion that PA improves physical and mental health in children of all abilities,^11^ yet less is known concerning participation levels and the direct benefits among children and adolescents with disabilities.^12^ In order to address this evidence gap, there is a need to establish surveillance systems to effectively monitor current levels of PA.^12^^,^^13^ The availability of PA estimates for children and adolescents with disabilities is of upmost importance to ensure policies can be developed and implemented effectively.^12^ The 2022 report card therefore included a set of grades specific to children and adolescents with disabilities, known as the para report Card.^13^

The aim of this study, produced as part of the Active Healthy Kids Global Alliance (AHKGA) Global Matrix 4.0, is to monitor changes and assign grades across a range of PA indicators for children and adolescents across the island of Ireland, including children and adolescents with disabilities, enabling important comparisons to be drawn. A review of the 2014^8^ and 2016^9^ report cards highlight that most indicators were awarded the same grade in 2014 and 2016, with improvements observed for ‘*Community and Environment’*, *‘Organised Sport and PA’* and *‘Overall PA’*. A report card was not produced for Ireland in 2018, due to a lack of new evidence from the 2016 iteration. This paper outlines the process, methods and outcomes of the Ireland North and South PA report card and para report card for children and adolescents 2022.

## 2 Methods

### 2.1 Procedure

The 2022 Research Working Group (RWG) was established in October 2020 and comprised academics from institutions across the island of Ireland, and representatives from the Institute of Public Health in Ireland. A sub-working group was established to focus on data for indicators disaggregated by disability.

Indicators and their benchmarking criteria are outlined in Supplementary File 1. For consistency, international standardised benchmarks were established and a grading scheme provided by the AHKGA.^14^ The RWG reviewed the available data for each indicator, and assigned a provisional grade based on factors including sample size, methodology and inequalities in the data. Following discussion and agreement, the RWG ‘proposed grades’ as per the standardised, international grading system (Table 1).

**Table 1:** Report Card Grades and their interpretation.

Grade	% meeting criteria	Interpretation
A+	94–100	We are succeeding with a large majority of children and youth
A	87–93	
A-	80–86	
B+	74–79	We are succeeding with well over half of children and youth
B	67–73	
B-	60–66	
C+	54–59	We are succeeding with about half of children and youth
C	47–53	
C-	40–46	
D+	34–39	We are succeeding with less than half but some children and youth
D	27–33	
D-	20–26	
F	<20	We are succeeding with very few children and youth
INC	Incomplete	Incomplete - insufficient or inadequate information to assign a grade

The Government indicator grade was determined using the Health-Enhancing PA Policy Audit Tool (HEPA PAT v2) and the scoring rubric developed by Ward and colleagues.^15^ An audit was carried out on all government policies published before August 2021 in both Ireland and Northern Ireland. Policies were scored on specific components of government policy related to supporting actions, accountable organisations, reporting structures, funding and monitoring and evaluation of policies. This allowed a % score to be applied to these areas of Government policy.

Proposed grades and accompanying rationale were circulated to stakeholder agencies for consultation. Ireland North and South report card grades were submitted to the AHKGA for audit in November 2021.

The para report card was a parallel project that used the same grading scheme and process as the AHKGA PA report cards,^13^ with further detail on data availability, methodology and grading process previously reported.^16^ Briefly, datasets were identified that included data disaggregated by disability, and disability was defined separately within each included study (Supplementary File 2). A SWOT (Strengths, Weaknesses, Opportunities and Threats) analysis was undertaken with representatives from national disability sport organisations to discuss and agree the grades.^16^ On completion of the stakeholder consultation and AHKGA audit, the grades were finalised and the report card was prepared.

### 2.2 Data sources, benchmarks, and grades

As Ireland did not participate in Global Matrix 3.0, academic articles and datasets available from 2016 up to March 2020 (i.e., prior to COVID-19 pandemic) were eligible for inclusion in the report card. Data collected since the implementation of COVID-19 public health measures in March of 2020 were excluded.

The following datasets formed the basis of the 2022 grades, but are supplemented, where highlighted, with additional studies and reports. No disability specific surveys were identified^16^; however, five surveys did include a measure for disability (Supplementary File 2).^16^

### 2.3 Northern Ireland datasets

#### 2.3.1 UK Millennium cohort study (MCS)^17^

The MCS is a longitudinal study that follows the lives of children born across the UK. Data from Wave 7 Northern Ireland sub-sample were included. Data were self-reported by the adolescent (n = 976, aged 17 years) from 2017-2019.^17^

#### 2.3.2 Young persons’ Behaviour and attitude survey (YPBAS)^18^

Results of the seventh round of this survey were included.^18^ Data were collected in 2019/2020 using a self-report questionnaire (n = 8,118, aged 11–16 years).

#### 2.3.3 Continuous Household Survey^19^

Data included in this report were collected in 2019/2020, with data on children's active transport (n = 898, aged 4–18 years) reported by a parent/guardian.^19^

### 2.4 Ireland datasets

#### 2.4.1 Growing Up in Ireland (GUI)^20^, ^21^, ^22^

Data from the Growing Up in Ireland (GUI) Cohort ’08 (formerly Infant cohort) and Cohort ’98 (formerly Child Cohort) were consulted. GUI is a longitudinal study in Ireland. Data for both cohorts were collected using questionnaires completed either by the primary caregiver, the child, or both. Data from Cohort ‘98 Wave 3 collected in 2015–2016 (n = 6,039, 17–18 years),^20^ Cohort ’08 Wave 4 collected in 2016 (n = 5,344, 7–8 years)^21^ and Cohort ‘08 Wave 5 collected in 2017/2018 (n = 8,032, 9 years)^22^ are reported.

#### 2.4.2 Health behaviour of school-aged children (HBSC)^23^

The HBSC study is undertaken every four years and aims to gain insight on young people's health and wellbeing in a social context in Ireland. Data collection took place in 2017/2018, using self-report questionnaires (n = 15,557, 8–18 years).^23^

### 2.5 All-island datasets

#### 2.5.1 Children's Sport Participation and Physical Activity Study (CSPPA)^3^

The CSPPA study is a cross-sectional study examining participation in sport, PA and PE among children aged 10–18 years. The 2018 study collected data on primary and post-primary level pupils (aged 10–18 years), with 6,651 pupils (n = 1,954 Northern Ireland, n = 4,697 Ireland) completing a self-administered questionnaire.^3^

## 3 Results

The 2022 Ireland North and South report card represents the third assessment of PA indicators for children and adolescents living on the island of Ireland. The grades assigned to each indicator and supporting evidence are shown in Table 2. Further detail on the supporting evidence and rationale for each grade for disability is presented elsewhere.^16^ Table 3 outlines the grade trends since 2014.

**Table 2:** Grades and rationales for Ireland's 2022 Report card.

Indicator	Grade	Rationale
Overall physical activity	C-	Of the eight data sources used to grade this indicator, six used self-reported (child) or proxy-reported measures. One used device measured (accelerometer) data alone^31^ and one used self-reported data and supported findings by device measured (accelerometer) data in a sub-sample of participants.^3^ Based on subjective measures, the proportion meeting the benchmark of 60 min of MVPA per day ranged from 8.4 %^17^ to 24.9 %.^23^ The proportion meeting the benchmark varied across studies using accelerometers; 11 % of 10–18-year-olds achieved 60 min per day (n = 275),^3^ while 56.7 % of 8–9-year-olds averaged 60 min of MVPA per day.^31^
Organised sport and physical activity	C	Five data sources (4 from Ireland, 1 from both jurisdictions) were consulted for this indicator, with all data sources using a self-reported measure. The CSPPA study^3^ outlined age-related differences in sport participation for both community-based sport and school sport. In Northern Ireland, 44 % of primary and 40 % of post-primary pupils participated in community sport at least twice a week. For school sport, the proportions were slightly higher; 51 % of primary and 44 % of post-primary. In contrast to Northern Ireland, the figures for school sport were lower compared with community sport in Ireland; 53 % of primary and 48 % of post-primary pupils reported participating in school sport, compared with 66 % of primary and 52 % of post-primary pupils participating in community sport two times per week.
Active Play	INC	This indicator was graded as *INC* in the absence of data which aligned with the benchmark. Five data sources were identified in the grading of this indicator. All data sources were from Ireland, and all used subjective measures (proxy, or self-report).^21^Data from the European Childhood Obesity Surveillance Initiative (COSI) highlighted that 43 % of parents reported their children played for about 2 h per day or more on weekdays, compared with 84.6 % on weekends (n = 1,263, 6–8-year-olds).^32^
Active Transportation	NI D-ROI D	Four data sources were identified, and all used self-reported measures. The CSPPA study highlighted a discrepancy in active travel across the island of Ireland, with 12.9 % of children in Northern Ireland usually actively travelling to and from school, compared with 28.3 % of children in the Republic (n = 6,651, 10–18-year-olds) (unpublished data from CSPPA 2018).^3^ A smaller study in Northern Ireland reported a higher rate than CSPPA, with approximately one-fifth of primary and post-primary schoolchildren normally travelling to and from school via active transport.^19^
Sedentary behaviours	C-	Four data sources were identified that aligned with the benchmark, measuring screentime via questionnaire. The CSPPA study outlined age-related trends in screentime, with higher levels of screentime in post-primary children across both jurisdictions. A total of 58.5 % and 63 % of primary schoolchildren met the guideline of <120 min per day in Northern Ireland and Ireland respectively, compared with 39.8 % and 42 % of post-primary aged pupils. Studies consistently highlighted higher screentime use at weekends compared with weekdays; data from GUI Wave 5 reported 85 % met the guideline of <2 h on weekdays, compared with just 49.5 % at weekends.^22^
Physical Fitness	INC	Nine data sources were identified for this indicator, however, only three followed the Eurofit procedure published by Tomkinson,^33^ which the benchmark was based on. Although 3 datasets did report outcomes that aligned with the Eurofit battery test,^3^^,^^34^^,^^35^ including cardiorespiratory fitness, upper and lower body muscular strength, and flexibility, the data reported in these papers did not align with the normative values needed to grade against the benchmark, and this indicator was graded as INC.
Family and peers	D+	Five data sources contributed to the grading of this indicator. Data from the Healthy Ireland Survey (Ireland only) reported approximately half of parents (49 %) met the PA guidelines (n = 2,001 parents).^36^ Data from the Irish Sports Monitor highlighted a lower proportion, with 32.3 % of parents meeting the guidelines (n = 2,596).^37^ Just over a third (36.5 %) of primary caregivers reported participating in sport/and or PA with their child at least once per week (n = 8,032, 9 year old children).^22^ Only one dataset provided evidence on the influence of peers; 44.1 % and 46.9 % of primary children in Northern Ireland and Ireland reported that their friends ‘sometimes’ encouraged them to do sport or physical activities, compared with 37.4 % and 44.7 % of post-primary schoolchildren.^3^
School	C-	Four data sources were used in grading this indicator, all were self-report, and all data was collected in Ireland. In terms of facilities available for sport and PA, data from GUI highlighted most teachers (63.3 %) reported the facilities in their school as ‘Good or Excellent’ (n = 4,728 teachers).^20^ All principals who participated in the COSI survey reported that their schools had an outdoor play area, with 77 % having access to an indoor gym (n = 131 principals).^32^ The Lifeskills survey, which sampled 1,796 schools found that 65 % of primary schools and 59 % of post-primary schools have a PA policy in place.^38^
Physical Education	D	Against the benchmark of the percentage of children and adolescents receiving the recommended amount of PE each week in school (2 h/week for post primary),^39^^,^^40^ this indicator was graded as *D,* and INC for disability in the absence of available data. The CSPPA study reported 40 % of post-primary schoolchildren in Northern Ireland and 23 % in Ireland met the recommendations.^3^
Community and Environment	B+	Six data sources were used in this grading, with four from Ireland, one from Northern Ireland and one from both jurisdictions. All data sources used self-report measures. Data from the HBSC study reported 61 % of children felt their local area had good places to spend their free time (n = 15,557 8-18-year-olds).^23^ Data from parents in the GUI study indicated that the majority (>90 %) agreed/strongly agreed it was safe for their children to play outside.^22^
Government	B	Following the HEPA PAT V2 and the scoring rubric outlined previously,^15^ the audit of Government policy and completion of the scoring rubric indicated a policy score of 67.5 %, equating to a *B* grade.

**CSSPA,** Children's Sport Participation and Physical Activity Study; GUI, Growing Up in Ireland; HEPA PAT, Health-Enhancing Physical Activity Policy Audit Tool INC, Incomplete; MVPA, Moderate-to-vigorous physical activity.

**Table 3:** Summary of grades awarded to each indicator for Irish Report Card 2014, 2016, 2022.

Indicator	2014	2016	2022	
			Main	Disability
Overall Physical Activity	D-	D	C-	F
Organised Sport and Physical Activity^a^	C-	C- (ROI)	C	D
		C+ (NI)		
Active Play	INC	INC	INC	INC
Active Transportation	D	D	NI D-	D-
			ROI D	
Sedentary Behaviours^b^	C-	C-	C-	C-
Physical Fitness^c^	–	–	INC	INC
Family and Peers^d^	INC	INC	D+	C
School	C-	D	C-	C-
Physical Education	D-	D-	D	INC
Community and Environment^e^	B	B+	B+	B-
Government^f^	INC	INC	B	B

INC: Incomplete.

a Previously known as ‘Organised Sport Participation’.

b Previously known as ‘Sedentary Behaviour: TV viewing’.

c Physical Fitness not previously included as an indicator.

d Previously known as ‘Home (Family)’.

e Previously known as ‘Community and Built Environment’.

f Previously known as ‘Government Strategies and Investments’.

**Fig. 1:**
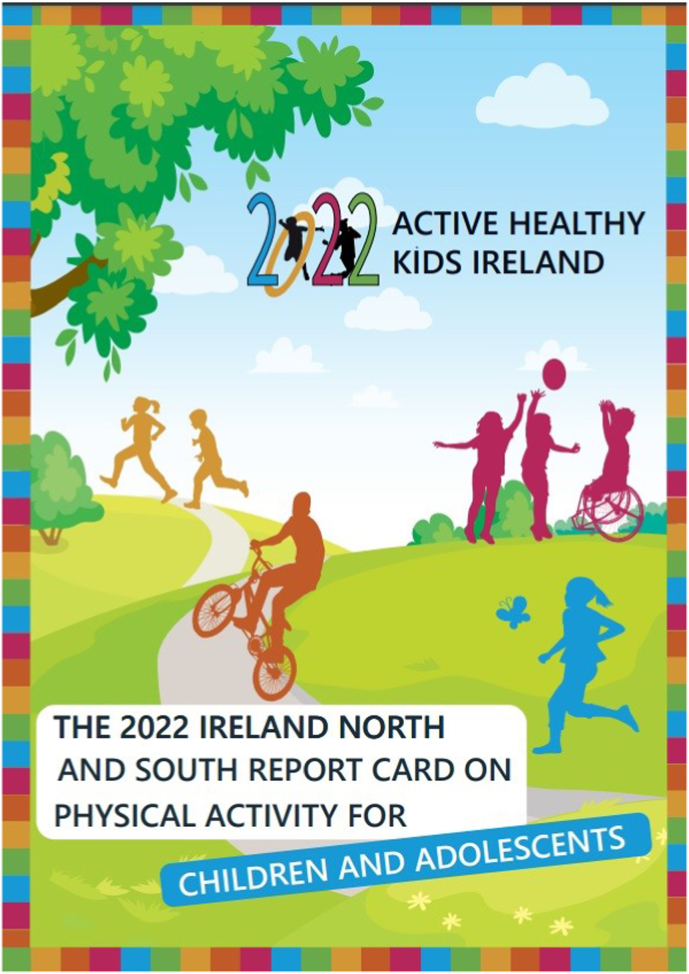
Front cover of the 2022 Ireland North and South Physical Activity Report Card.

A short-form and long-form report card (Fig. 1) were produced as part of the Report Card process.

## 4 Discussion

Within Global Matrix 4.0, Ireland was assigned an overall grade C- (behavioural indicators average D+, sources of influence average C+).^24^ When compared with the average grades for countries across the Anglosphere geo-cultural region (Overall grade D+, behavioural indicators average D, sources of influence average C),^24^ Ireland has been graded just above the average across the indicators. These international comparisons, alongside the small progress that has been achieved in relation to several indicators, namely ‘*Overall PA’, ‘School’* and ‘*PE’* indicate that we are making some positive steps. Disability-specific grades, which often were graded below the overall dataset, highlights that much work is still needed to promote PA participation for all children and adolescents. In addition, limitations with the availability of surveillance data for children and adolescents highlight the importance of further monitoring and evaluation of PA participation.

The grade for ‘**Overall PA’ (C-)** represents an improvement from 2016 (Grade D) but may be attributed in part to change in the benchmark and not reflective of an increase in the proportion of children and adolescents meeting the guidelines. Several issues were identified in the measurement of this indicator; there are still inconsistencies in how PA is measured across surveillance studies, which limits comparisons with both the AHKGA benchmark, and the national PA guidelines. In addition, the benchmark does not consider other aspects of the PA guidelines, for example, muscle strengthening exercises, with adherence to these components of the guidelines not captured or reflected in the grading. Inequalities in the data are demonstrated by a grade **F** for disability, highlighting the need for both further efforts to target this population, and better inclusivity of this population in research.

**‘Organised sport and PA’** was graded C (Disability D). The distinction between community sport and school sport club participation is important for the implementation, funding, and monitoring of sports facilities, however only one study collected data in this way,^3^ limiting comparisons across different settings. Further research employing direct observation would help distinguish participation in organised sport from other domains of PA, and may in turn provide more robust data across a number of indicators. ‘**Active Play’ (INC)** continues to be graded as INC due to a lack of consensus on a definition of Active Play; coupled with a lack of standardised tools to measure and quantify this behaviour. Response options within existing surveys do not align with the benchmark, contributing to a dearth of data to support a grade for this indicator. Furthermore, there is a lack of evidence using direct and device-based measures*,* which are important to minimise bias which may occur by a reliance on subjective measures, given the challenges with misinterpreting play as other forms of PA.^25^

The D grade for ‘**Active Transportation’** has remained consistent for the Ireland since 2016 and decreased slightly for Northern Ireland from a D in 2016 to D-in 2022. Similar with other indicators, comparisons across previous iterations of the report card are hampered by a change in the benchmark. The revised benchmark, which grades the indicator based on ‘active transportation to AND from places’, rather than ‘OR’ has had an impact on both the availability of data sources that align with the benchmark, and the proportion of youth meeting this benchmark. Although not used for grading (based on the benchmark) these data provide important insights into how variable active transport behaviours can be; for example, the YPBAS reported that a higher proportion of young people walk home from school (22 %), compared with to school (16 %).^18^ In order to fully understand the potential to improve the **D-**grade for disability and target those who could increase their levels of active travel, further disaggregation of data on impairment types is needed.

There has been no change in the grade for ‘**Sedentary Behaviour’ (C-)**, with the same grade awarded for disability. The challenge with the measurement of this indicator, as highlighted previously,^9^ is that self-reported screen-time may not be an appropriate surrogate measure of overall sedentary behaviour. Furthermore, the current guidelines against which the benchmark is set does not provide a time limit for non-screen related sedentary behaviours. The evidence base for sedentary behaviours across the island of Ireland is drawn largely from self-report data, and there is inconsistency in the measurement of screen time across studies. There is likely to be an overlap in behaviours also, which presents challenges for measurement, as some internet use may be television viewing (e.g., watching videos on Netflix and/or YouTube) and often behaviours may be taking place simultaneously (for example, an individual may use the internet while watching television, therefore data should be interpreted with caution.

Despite an increase in research outputs in this area, ‘**Physical Fitness’** was graded as INC due to challenges aligning available data with the benchmark. To the authors' knowledge, there has been no specific data published that report outcomes aligned with the benchmark. Skills-related measures (for example, agility, speed, balance) are particularly underreported in studies across the island of Ireland. The lack of data aligned with the benchmark was not unique to our Report card, with more than half of countries in Global Matrix 4.0 unable to assign a grade for this indicator.^24^ A small improvement was observed overall for ‘**PE’ (D)** however a lack of available data specific to children and adolescents with a disability meant this indicator was graded INC for disability. The Ireland North and South report card was the only country to assign a separate grade to PE^24^; the ‘**School’** indicator covers PE but focuses on the percentage of schools as opposed to the percentage of pupils. Given the importance of PE, not just to overall PA levels, but to building the confidence, competencies, knowledge, motivation and understanding associated with physical literacy,^26^ we felt this warranted a separate focus and grade. Reform in the curricula supporting delivery of PE has moved in a positive trajectory across the island but the implementation of these needs to be considered and monitored. PE is well established in the post-primary curricula across both jurisdictions, as a compulsory subject delivered by specialist teachers. The data, however, indicates not all schools are providing the recommended levels of PE for children and adolescents.

Looking to the grades for sources of influence, **Family and Peers (D+, disability C)** was assigned a grade for the first time. The availability of data stems mostly from adult based surveys, where it was possible to distinguish between non-parents and parents (having at least one child under 18 years). This is a broad age range and different responses may be evident if parents of younger and older children were examined separately. Limited data is available for the influence of peers on PA and should be a focus in future studies. **Community and Environment (B+)** continues to be the highest graded indicator and has remained consistent since 2016. Disability was graded lower at B-, with stakeholders recognising the current focus on increasing the provision of accessible playgrounds across the island.^16^ It is worth highlighting that most data available focuses on perceptions of safety, and we know less about how PA is encouraged within community spaces. As with other indicators, studies that employ device-based measures, for example GPS, would be welcomed to provide more understanding of the communities and/or environments where children and young people live and/or participate in PA.

Although a steady improvement has been observed for **School (C-)** since the first report card in 2014, the grades cannot be directly compared due to change in the benchmark used for this indicator. The School indicator was one of few indicators within the Report Card to receive the same grading for children and adolescents with disability. It is encouraging to see the proportion of schools with a PA plan, as this is a key part of the whole-of-school approach to promote PA.^27^ In Ireland, the Active School Flag initiative provides schools with relevant policies to promote an active school.^28^ More recently, there have been provisions for the Active School Flag in special schools in Ireland, however this was not captured within the reporting timeframe for this Report Card. In addition, such a national programme has yet to be reported in Northern Ireland.

Finally, **Government (B)** was assigned a grade for the first time, with Ireland, alongside 11 other countries^24^ using the HEPA PAT v2 methodology. There have been some significant developments for all-island PA policy including the launch of a new 10-year Sport and PA Strategy in March 2022 for Northern Ireland.^7^ The strategy has identified several goals to address barriers to inclusive sport and PA, including improving physical literacy and promoting inclusion through community engagement.^7^ In Ireland, an implementation review of the 2016 National PA Policy provided policy considerations to support children and adolescents. These included the development of peer-led interventions for adolescents and family-based programmes for children. Other national policy-level changes have included the expansion of the National Transport Authority investment to develop active travel walking and cycling infrastructure across Ireland by 2025. Close monitoring and evaluation of these policies will be needed to understand the impacts for children and young people.

One of the primary strengths of the 2022 edition was the inclusion of representatives in the RWG from across the island of Ireland, which allows for continued cross-border cooperation in PA advocacy. Addressing gaps in the 2016 Report Card was a priority for the RWG, and the inclusion of disability specific grades is a notable strength of this 2022 Report Card, as these groups tend to be underrepresented.^29^ The insights provided by the grading of data specific to children and adolescents with disabilities highlights the lower levels of PA in this group and provides a call to action to ensure that monitoring and surveillance of PA in this group should be a priority for research. Ireland is at the forefront of this work, producing a separate set of grades alongside 14 other countries that participated in GM 4.0,^14^ and one of only a handful of countries to present the disability grades within our main country Report Card release. The RWG are also encouraged by the availability of new data sources which have collated data on an all-island basis.^3^

Although grades are based on the best available data, significant research gaps remain unaddressed. In some instances, the availability of appropriate data is limited by the selected benchmarks. This was apparent for physical fitness, for example, where we have emerging research but could not use as the reference points did not align with the harmonised methodology of GM 4.0. In most instances, however, the issue is clear gaps in the research. There is limited data available for Active Play, and an over-reliance on self-reported measures across several indicators. There continues to be a gap in terms of availability of data between jurisdictions, with greater data available for Ireland. There is no available data on active play or physical fitness specific to Northern Ireland, for example. Most data used to assign grades is based on older children (8 years and above) and adolescents, therefore younger children are underrepresented.

## 5 Conclusions

The 2022 Ireland North and South Report Card on PA demonstrates that fewer than 20 % of children and adolescents with disabilities are meeting the current PA recommendations, with less than half of children and adolescents achieving the guideline of <2 h of screentime/day. Although some progress has been made across a few indicators since 2016, caution is warranted when it comes to interpreting the grades across time points and indeed across countries, owing to variations in the benchmarks used and the availability of self-report and device-based measures of PA behaviours. The number of challenges identified by the Ireland report card are not unique,^24^ and therefore there is a need for all participating countries in GM 4.0 to share knowledge and experience to strengthen the report card process.

As with most countries participating in GM 4.0,^24^ grades were informed by evidence pre-COVID-19 pandemic. With evidence already showing the impact of COVID-19 restrictions on PA levels in children and adolescents,^30^ the next report card will provide an important marker on the impact across the island of Ireland, and whether the pandemic has worsened or reduced the inequities identified in the present report card.

The key recommendations from the 2022 Report Card are: (1) Progress the development of a framework for the systematic surveillance of indicators related to PA for children and adolescents, including those with disabilities; (2) Address persistent gaps in data availability in relation to a number of indicators, for example, ‘Active Play’ and for some sub-groups of children and adolescents, for example, data in younger children; (3) Continue to develop policy measures that address inequalities highlighted in the report across a range of determinants including disability, gender, socioeconomic status, and age that impact on children and adolescent PA levels.

## Funding source

This work was supported by funding from the 10.13039/501100001626Public Health Agency (Northern Ireland), 10.13039/100018754Department of Health (Ireland) and Healthy Ireland, Sport Northern Ireland, Sport Ireland, and the Institute of Public Health in Ireland. The Institute of Public Health is jointly funded by the Departments of Health in Ireland and Northern Ireland. The funders had no role in the design of the study. The Institute of Public Health were part of the research working group (represented by two staff members) and were involved in the grading of the data and writing of the manuscript. All other funders had no role in the collection, analyses, or interpretation of the data, or in the decision to publish the results.

## Author statement

Conceptualization, Angela Carlin, Sarahjane Belton, Deirdre Harrington, Helen McAvoy and Marie Murphy; Formal analysis, Angela Carlin, Sinead Connolly, Tamsyn Redpath, Sarahjane Belton, Tara Coppinger, Conor Cunningham, Alan Donnelly, Kieran Dowd, Deirdre Harrington, Elaine Murtagh, Kwok Ng, Lauren Rodriguez, Catherine Woods, Helen McAvoy and Marie Murphy; Funding acquisition, Angela Carlin, Sarahjane Belton, Lauren Rodriguez, Helen McAvoy and Marie Murphy; Investigation, Angela Carlin, Sinead Connolly, Tamsyn Redpath, Sarahjane Belton, Tara Coppinger, Conor Cunningham, Alan Donnelly, Kieran Dowd, Deirdre Harrington, Elaine Murtagh, Kwok Ng, Lauren Rodriguez, Catherine Woods, Helen McAvoy and Marie Murphy; Methodology, Angela Carlin, Sinead Connolly, Tamsyn Redpath, Sarahjane Belton, Tara Coppinger, Conor Cunningham, Alan Donnelly, Kieran Dowd, Deirdre Harrington, Elaine Murtagh, Kwok Ng, Lauren Rodriguez, Catherine Woods, Helen McAvoy and Marie Murphy; Project administration, Angela Carlin, Sinead Connolly, Tamsyn Redpath, Lauren Rodriguez, Helen McAvoy and Marie Murphy; Resources, Angela Carlin, Sinead Connolly, Tamsyn Redpath, Sarahjane Belton, Tara Coppinger, Conor Cunningham, Alan Donnelly, Kieran Dowd, Deirdre Harrington, Elaine Murtagh, Kwok Ng, Lauren Rodriguez, Catherine Woods, Helen McAvoy and Marie Murphy; Writing – original draft, Angela Carlin, Sinead Connolly, Tamsyn Redpath and Marie Murphy; Writing – review & editing, Sarahjane Belton, Tara Coppinger, Conor Cunningham, Alan Donnelly, Kieran Dowd, Deirdre Harrington, Elaine Murtagh, Kwok Ng, Lauren Rodriguez, Catherine Woods and Helen McAvoy.
