# Supplementary material for: Results from Ireland North and South’s 2022 report card on physical activity for children and adolescents
Source: J Exerc Sci Fit. 2023 Dec 9;22(1):66–72. doi: 10.1016/j.jesf.2023.12.003 (PMC10762465; doi:10.1016/j.jesf.2023.12.003)
Supplement: Multimedia component 1 [file mmc1.docx]

**Supplementary File 1 Active Healthy Kids Global Alliance Global Matrix and benchmark criteria used for the Physical Activity Report Card**

| **Indicator** | **AHKGA Definition** | **AHKGA criteria** |
| --- | --- | --- |
| Overall physical activity | Any bodily movement produced by skeletal muscles that requires energy expenditure. | - % of children and adolescents who meet the Global Recommendations on Physical Activity for Health, which recommend that children and youth accumulate at least 60min of moderate-to vigorous-intensity physical activity per day on average. - OR % of children and youth meeting the guidelines on at least 4d a week (when an average cannot be estimated). |
| Organised sport and physical activity | A subset of physical activity that is structured, goal-oriented, competitive and contest-based. | - % of children and adolescents who participate in organized sport and/or physical activity programs. |
| Active play | Active play may involve symbolic activity or games with or without clearly defined rules; the activity may be unstructured/unorganized, social or solitary, but the distinguishing features are a playful context, combined with activity that is significantly above resting metabolic rate. Active play tends to occur sporadically, with frequent rest periods, which makes it difficult to record. | - % of children and adolescents who engage in unstructured/ unorganized active play at any intensity for more than 2 h a day. - % of children and adolescents who report being outdoors for more than 2 h a day. |
| Active transportation | Active transportation refers to any form of human-powered transportation – walking, cycling, using a wheelchair, in-line skating or skateboarding. | - % of children and adolescents who use active transportation to get to and form places e.g. school, park, mall, friend’s house |
| Sedentary behaviours | Any waking behaviour characterized by an energy expenditure ≤1.5 metabolic equivalents, while in a sitting, reclining or lying posture. | - % of children and adolescents who meet the Canadian Sedentary Behaviour Guidelines (5- to 17-y-olds: no more than 2 h of screen time per day). |
| Physical fitness | Characteristics that permit a good performance of a given physical task in a specified physical, social, and psychological environment | - Average percentile achieved on certain physical fitness indicators based on the normative values published by Tomkinson et al. ^33^ ^33^ |
| Family and peers | Any member within the family who can control or influence the physical activity opportunities and participation of children and youth in this environment. | - % of family members (such as parents or guardians) who facilitate physical activity and sport opportunities for their children (e.g., volunteering, coaching, driving, paying for membership fees and equipment). - % of parents who meet the Global Recommendations on Physical Activity for Health, which recommend that adults accumulate at least 150 min of moderate-intensity aerobic physical activity throughout the week or do at least 75 min of vigorous-intensity aerobic physical activity throughout the week or an equivalent combination of moderate- and vigorous-intensity physical activity. - % of family members (e.g., parents, guardians) who are physically active with their kids - % of children and adolescents with friends and/or peers who encourage and support them to be physically active - % of children and adolescents who encourage and support their friends and/or peers to be physically active |
| School | Any policies, organisational factors (e.g., infrastructure, accountability for policy implementation) or student factors (e.g., physical activity options based on age, gender or ethnicity) in the school environment that can influence the physical activity opportunities and participation of children and youth in this environment. | - % of schools with active school policies (e.g., daily physical education (PE), daily physical activity, recess, “everyone plays” approach, bike racks at school, traffic calming on school property, outdoor time). - % of schools where the majority (≥80%) of students are taught by a PE specialist. - % of schools where the majority (≥80%) of students are offered the mandated amount of PE. - % of schools that offer physical activity opportunities (excluding PE) to the majority (>80%) of their students. - % of parents who report their children and youth have access to physical activity opportunities at school in addition to PE classes. - % of schools with students who have regular access to facilities and equipment that support physical activity (e.g., gymnasium, outdoor playgrounds, sporting fields, multipurpose space for physical activity, equipment in good condition). |
| Community and environment | Any policies or organisational factors (e.g., infrastructure, accountability for policy implementation) in the municipal environment that can influence the physical activity opportunities and participation of children and youth in this environment. | - % of children or parents who perceive their community/municipality is doing a good job at promoting physical activity (e.g., variety, location, cost, quality). - % of communities/municipalities that report they have policies promoting physical activity. - % of communities/municipalities that report that they have infrastructure geared towards promoting PA - % of children and/or parents who report having facilities/ programs/ parks/ playgrounds available to them in their community - % of children and/or parents who report having well-maintained facilities/ parks/playgrounds in their community that are safe to use. |
| Government | Any governmental body with authority to influence physical activity opportunities or participation of children and youth through policy, legislation or regulation. | - Evidence of leadership and commitment in providing physical activity opportunities for all children and youth. - Allocated funds and resources for the implementation of physical activity promotion strategies and initiatives for all children and youth. - Demonstrated progress through the key stages of public policy making (i.e., policy agenda, policy formation, policy implementation, policy evaluation and decisions about the future). - HEPA PAT v2 and the scoring rubric published by Ward et al. ^15^ |

AHKGA; Active Healthy Kids Global Alliance

**Supplementary File 2 Data sources used for grading indicators for children and adolescents with disability, including measure of disability and estimated prevalence**

| Study | Jurisdiction | Measure of disability (as defined by study) | Estimated disability prevalence |
| --- | --- | --- | --- |
| Growing Up in Ireland Infant Cohort Wave 5 ^22^  (n=8,032, 9 years) | Ireland | 1. Does your child have any longstanding illness, condition or disability? (Parent report) | 4.0% |
| Growing Up in Ireland Child Cohort Wave 3 ^20^  (n=6,039, 17-18 years) | Ireland | 1. Do you have any particular special educational need or disability that affects/affected your learning while at post-school education or training (other than ‘exceptionally able’ or ‘gifted’)? (Self-report)  2. Does your child have any of the following long-lasting conditions or difficulties? Blindness, Deafness, Daily activities, Intellectual disability, Learning difficulties, Psychological, Pain or Breathing, Other. (Parent report) | 5.8% |
| Health Behaviour of School-aged Children ^23^  (n=15,557, 8-18 years) | Ireland | 1. Long-term illnesses or Disabilities as diagnosed by a doctor (Self-report) | 12.3% |
| Irish Sports Monitor  (n=570, 16-18 years) ^30^ | Ireland | 1. Long-term illness or disability that limits daily activities (Self-report) | 12.1% |
| Young Persons’ Behaviour and Attitude Survey  (n=8,118, 11-16 years) ^18^ | Northern Ireland | 1. Physical or mental health conditions or illnesses, lasting or expected to last, for 12 months or more? (Self-report) | 18.7% |
| Children’s Sport Participation and Physical Activity Study  (n=6,651, 10–18 years) ^3^ | Both | 1. Washington Group/UNICEF Child Functioning Module – self-report version. 11 items on difficulties in core functions (seeing, hearing, walking, talking, handling objects, remembering, concentration, learning, changes to routines, controlling behaviour, and getting friends). “A lot of difficulties” or “cannot do” were coded as a person with disabilities. | 15.0% |
